# Proprietary tomato extract improves metabolic response to high-fat meal in healthy normal weight subjects

**DOI:** 10.3402/fnr.v60.32537

**Published:** 2016-10-04

**Authors:** Xavier Deplanque, Delphine Muscente-Paque, Eric Chappuis

**Affiliations:** Naturalpha SAS, Parc Eurasanté, Loos, France

**Keywords:** cardiovascular, carotenoid-rich tomato extract, oxidative stress, tomato, oxidized LDL

## Abstract

**Background:**

Low-density lipoprotein (LDL) oxidation is a risk factor for atherosclerosis. Lycopene and tomato-based products have been described as potent inhibitors of LDL oxidation.

**Objectives:**

To evaluate the effect of a 2-week supplementation with a carotenoid-rich tomato extract (CRTE) standardized for a 1:1 ratio of lycopene and phytosterols, on post-prandial LDL oxidation after a high-fat meal.

**Design:**

In a randomized, double-blind, parallel-groups, placebo-controlled study, 146 healthy normal weight individuals were randomly assigned to a daily dose of CRTE standardized for tomato phytonutrients or placebo during 2 weeks. Oxidized LDL (OxLDL), glucose, insulin, and triglyceride (TG) responses were measured for 8 h after ingestion of a high-fat meal before and at the end of intervention.

**Results:**

Plasma lycopene, phytofluene, and phytoene were increased throughout the study period in the CRTE group compared to placebo. CRTE ingestion significantly improved changes in OxLDL response to high-fat meal compared to placebo after 2 weeks (*p*<0.0001). Changes observed in glucose, insulin, and TG responses were not statistically significant after 2 weeks of supplementation, although together they may suggest a trend of favorable effect on metabolic outcomes after a high-fat meal.

**Conclusions:**

Two-week supplementation with CRTE increased carotenoids levels in plasma and improved oxidized LDL response to a high-fat meal in healthy normal weight individuals.

Aerobic metabolism generates reactive oxygen species (ROS) including superoxide anion, hydrogen peroxide, and hydroxyl radicals. Biological and physiological processes are regulated by ROS that act as signaling molecules, allowing environmental adaptations of mammalian cells; however, ROS also interact with different biological targets and are commonly associated with oxidative stress linked to the development of certain pathologies where lipids, proteins, and DNA damage might be involved (1). ROS have a causal role in atherosclerosis that consists of migration, accumulation, and proliferation of foam cells in intimal smooth muscle cell, leading to the development of fibrofatty plaque sclerosis and cardiovascular events. An understanding of the underlying mechanisms involved in atherogenesis has dramatically improved over recent years and has revealed that endothelial dysfunction, the first step in this process, is a consequence of decreased nitric oxide (NO) bioavailability and increased generation of ROS in the vascular wall. Circulating low-density lipoproteins (LDLs) are entrapped in the subendothelial space of the vascular endothelium and subjected to oxidative modification to form oxidized LDL (OxLDL) particles that are internalized by macrophages and accumulate in foam cells, leading to atherogenesis (2). Absorption of exogenous antioxidants such as ascorbate (vitamin C) and α-tocopherol (vitamin E) has been proposed as a strategy to inhibit atherogenesis through restoration of NO bioavailability and ability to limit LDL oxidation, but promising results from small trials have not been confirmed in large interventional trials (2). Other antioxidants such as lycopene, the strongest oxygen quencher amongst carotenoids, have a protective role toward cardiovascular diseases (3) and demonstrate an *in vitro* ability to protect lipoproteins from oxidation, but *in vivo* evidence is still conflicting (4, 5). Results of interventional trials suggest that lycopene alone is less effective than whole tomatoes or complex tomato extracts in improving cardiovascular risk factors linked to oxidative stress such as OxLDL (5, 6).

Carotenoid-rich tomato extract (CRTE) is a tomato extract displaying health benefits similar to that of whole tomato consumption. For example, evidence suggests that this complex improves blood pressure in never-treated grade I hypertensive subjects and in treated, but uncontrolled, hypertensive subjects (7, 8).

Administration of high-fat meals has been proposed as a challenge model for atherogenesis because it results in a postprandial (fed-state) response characterized by hypertriglyceridemia (9, 10), inducing an increase of plasma concentrations of OxLDL (11, 12), as well as other inflammatory and metabolic parameters (13–18).

Given the metabolic effects of tomato-based products, the aim of the present study was to investigate the effect of chronic administration of CRTE on postprandial OxLDL response after consumption of a high-fat meal challenge in healthy adults.

## Methods

### Participants

In total, 150 healthy males and females were recruited for this randomized, double-blind, parallel-groups, placebo-controlled study. Participants were required to be aged 18–70 years, with normal weight defined as body mass index (BMI) ≥18.5 and <25 kg/m^2^, plasma LDL cholesterol (LDLc) ≥100 and <220 mg/dL, plasma fasting triglycerides (TGs) <340 mg/dL, and plasma fasting glycemia (FG) <126 mg/dL; absence of type 1 and type 2 diabetes and cardiovascular disease or stroke and significant gastrointestinal disease; and without history of cancer or bariatric surgery. Subjects taking drugs or supplements that altered blood lipid levels as well as smokers and subjects consuming more than one alcoholic drink daily were excluded.

This monocentric study took place at the Naturalpha Clinical Nutrition Center, Hôpital Saint Vincent de Paul, Lille, France, and was conducted in accordance with the Declaration of Helsinki and the (2008 revisions), International Conference on Harmonization Harmonized Tripartite Guideline for Good Clinical Practice and the laws and regulations of Israel and France. The study received approval from Ethics Committee and French Competent Authority (Agence Nationale de Sécurité du Médicament; permission no. ID-RCB 2013-A00346-39), and all participants signed a written informed consent form before enrollment. CONSORT guidelines 2010 were considered.

### Intervention and study design

Subjects were selected during a screening visit (V1) up to 3 weeks before the baseline visit (V2), and eligible subjects were randomly assigned to take CRTE or placebo soft gel capsules. The treatment capsules were standardized for the levels of several phytonutrients such as lycopene and phytosterols in a 1:1 ratio (15 mg), as well as tocopherols and other tomato carotenoids, i.e. phytoene, phytofluene (4 mg), and β-carotene (0.5 mg). The capsules were taken once daily with the main meal (preferably lunch) for 2 weeks between V2 and the final visit (V3). During the intervention, participants received lifestyle and dietary recommendation aimed at controlling lycopene and tomato-based products intake.

At V2 and V3, blood was withdrawn to evaluate plasma lycopene, phytoene, and phytofluene baseline levels (at V2) and bioavailability and compliance (at V3). Then, subjects were served a high-fat breakfast (850 kcal; 50% of energy from fat; standardized in lycopene) that was ingested within 20 min. After ingesting the high-fat meals, blood was withdrawn during 8 h (except for insulin, 2 h) to evaluate efficacy (plasma OxLDL, glucose, TG, and insulin levels) of the intervention. Safety was evaluated by blood tests, a physical examination, vital signs, and reporting of adverse events (AEs). Compliance was assessed by collecting capsules at V3.

### Randomization and blinding

Randomization was stratified according to gender. Random sequence was generated by an independent statistician and subjects were enrolled by a Clinical Nutrition Center investigator/staff. Study products (CRTE and placebo) were provided in a bottle containing the capsules, bearing a product number that was assigned to the subject's randomization number at V2. Neither the subject nor the investigator/staff knew the composition of the product.

### Data collection

Physical examination, vital signs, AEs, and concomitant use of medication were recorded at each visit. Food recommendations were provided at each visit. Demographic data, medical history review, height and weight, and waist circumference were collected at V1. Safety parameters were collected at V1 and V3. Dietary instructions were provided to study participants at V1 and V2. Efficacy parameters were collected at V2 and V3, except for insulin (only at V3).

Blood collection for determination of plasma glucose and TGs was performed at 0, 30, 60, 90, 120, 240, 360, and 480 min after a high-fat meal. Blood collection for OxLDL was performed at 0, 180, 240, 360, and 480 min after high fat meal. Blood collection for insulin was performed at 0, 30, 60, 90, 120 and 180 min after high-fat meal.

### Biochemical measurements

After collection, blood samples were immediately transferred into cooled test tubes (at approximately +4°C) preloaded with EDTA (K3) and vortexed to ensure that all blood came into contact with the test tube wall. Samples were centrifuged within 15 min of sampling in a cooled centrifuge (at approximately +4°C). The supernatant (plasma) was divided into two aliquots of approximately equal volume, and the aliquots were transferred to a deep-freezer and stored at −80°C.

OxLDL was measured using an oxidized LDL ELISA kit (catalog no. 10-1143-01, Mercodia, Uppsala, Sweden). Quantitative determination of insulin was performed with an immunoassay method (catalog no. 33410, Beckman Coulter, Fullerton, CA), total TG with TG glycerol phosphate oxidase reagent (catalog no. 44580, Beckman Coulter), and plasma glucose with glucose reagent (catalog no. B24985, Beckman Coulter).

Lycopene, phytofluene, and phytoene in human plasma were determined with ultra-performance liquid chromatography with a C_18_ reverse phase column at multiple wavelengths by using photodiode array detector. For each sample, 500-µL plasma aliquots were spiked with 20 µL of an internal standard solution containing 20 µg/mL astaxanthin in reconstitution solution (100 µg/mL 2,6-di-*tert*-butyl-4-methylphenol [BHT] in isopropanol). Then, 500 µL of ethanol was added to the plasma by vortex for 5 sec followed by 4.0 mL of extraction solution (100 µg/mL BHT in hexane:dichloromethane, 80:20). The sample was then vortexed for 10 sec, sonicated for 5 min, and vortexed again for 5 sec. Samples were then centrifuged for 10 min at 4,000 rpm at room temperature, and 3.0 mL from the upper layer was transferred to evaporation tubes and dried in a SpeedVac trap under dark conditions for up to 50 min. A 500-µL aliquot of diethyl-ether was used to rinse down the dried sample from the walls of the evaporation tube to its base and subsequently dried in a SpeedVac trap under dark conditions for up to 20 min. The dried sample was then reconstituted in 200 µL of reconstitution solution, and 150 µL of the upper layer was transferred to autosampler vial with a glass insert.

### Statistical analyses

The primary objective of the study was to evaluate the effect of the CRTE on the reduction of postprandial LDL oxidation in healthy weight men and women. Based on the previous work of Burton-Freeman (19), sample size was calculated to detect a decrease of −3.0 units in OxLDL between the CRTE and placebo groups, with 90% power and an α level of 0.05, assuming that the common standard deviation for OxLDL is 5.0 units.

Statistical analyses were performed by Medistat with SPSS SAS version 9.1 (SAS Institute, Cary, NC).

OxLDL changes from baseline as well as relative changes (%) on each time point were analyzed using the paired t-test or signed rank test for two means (paired observations), as appropriate, within study arms. Comparative analysis between active and placebo group at any time point was performed using the analysis of variance (ANOVA) model for repeated measurements or the mixed-effect model for repeated measures (MMRM), including the fixed effect time and product and adjusted for baseline measurement and for additional covariates (such as sex) as appropriate. Changes from baseline at every collected time point until 480 min within group in glucose, TGs, and insulin blood levels were analyzed using the paired t-test or signed rank test (as appropriate). Comparative analyses of the changes at all times between groups were analyzed using repeated measurements ANOVA or MMRM. Differences in OxLDL, blood glucose, and TGs at V2 and V3 for every time point after the high-fat meal were analyzed using the paired t-test. Area under the curve (AUC_0–480_) was calculated over time for glucose, TGs, and insulin measurements. The changes from V2 to V3 in lycopene, phytofluene, and phytoene levels were analyzed using the paired t-test or signed rank test (as appropriate).

The intent-to-treat population included all randomized subjects who, as documented before the breaking of the study blind, (1) were dosed with the study product (either CRTE or placebo) and provided a baseline assessment and (2) provided at least part of the efficacy assessment (during the study day visit).

All tests were two tailed. A *p* value of 0.05 or less was considered statistically significant.

## Results

### Study flow, compliance, and adverse effects

One hundred and fifty subjects were enrolled in the study, and 146 completed the intervention between June and September 2013. One and three subjects withdrew from the CRTE and placebo groups, respectively.

Treatment compliance was similar in both study arms. An average of 6.2±2.1 capsules was returned in the CRTE arm and an average of 6.6±2.6 capsules was returned in the placebo arm.

CRTE was safe and well tolerated. No serious AEs occurred during the study. None of the subjects in the CRTE arm were discontinued from the study due to AEs, and none of the AEs led to an intervention or any significant additional concomitant therapy. No significant laboratory abnormalities or clinically significant changes in vital signs or physical findings were reported during the study.

### Baseline characteristics of participants

Mean age of participants was 34.8±11.7 and 34.9±11.4 years in the CRTE and placebo groups, respectively. Females represented 55.3% of the CRTE group and 55.4% of the placebo group. Mean BMI was 22.4±2.2 and 22.5±2.2 kg/m^2^ and mean waist circumference was 81.9±7.6 and 81.9±8.6 cm in CRTE and placebo groups, respectively.

### Bioavailability

Serum levels of lycopene, phytofluene, and phytoene at V2 and V3 are displayed in Table 1. At V2, plasma lycopene, phytofluene, and phytoene levels were similar between groups. Between V2 and V3, plasma lycopene, phytofluene, and phytoene levels significantly increased in the CRTE group compared to the placebo group.

**Table 1:** Serum levels of lycopene, phytofluene, and phytoene during the study

	Placebo			CRTE			
			
	*n*	Mean±SEM	p* within treatment	*n*	Mean±SEM	p* within treatment	p** between treatments
Lycopene (ng/mL)							
V2	72	430.5±12.8		75	404.6±13.7		0.1634
V3	71	412.5±14		75	501.4±5.3		<0.0001
Change from V2 to V3		−16.8±11	0.0922		96.8±13.1	<0.0001	<0.0001
Phytofluene (ng/mL)							
V2	72	153.9±9.1		75	148.6±9.6		0.4663
V3	71	150.0±9.4		75	284.1±14.1		<0.0001
Change from V2 to V3		−5.1±6.0	0.3190		135.5±11.1	<0.0001	<0.0001
Phytoene (ng/mL)							
V2	72	56.5±1.4		75	58.9±2.4		0.8792
V3	71	57.8±2.0		75	103.8±4.7		<0.0001
Change from V2 to V3		1.2±2.0	0.7741		45.0±4.5	<0.0001	<0.0001

SEM, standard error of the mean; V, visit.

* *p* by signed rank test indicates the statistical significance of the change within group.

** *p* by Wilcoxon test indicates the statistical significance of difference in the changes between groups.

### OxLDL levels after high-fat meal challenge

**Fig. 1:**
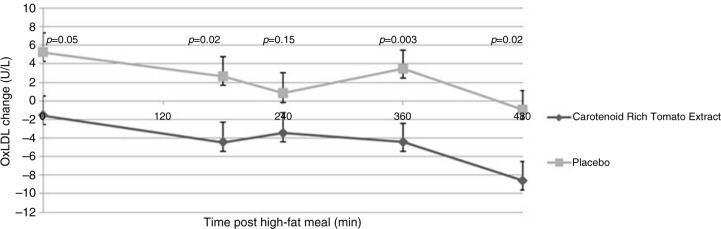
Change of postprandial OxLDL levels from baseline at V2 to V3 by time points and treatment (mean±SEM).

Changes in OxLDL levels after high-fat meal between V2 and V3 at different time points are illustrated in Fig. 1, and absolute values are reported in Table 2. At V2 and V3, no significant difference was noted between treatments at any time point after high-fat meal ingestion for absolute OxLDL values (Table 2). However, both treatments induced significant reductions in OxLDL levels for up to 6 h (360 min) after the high-fat meal. At the end of the intervention (V3), OxLDL levels were lower in the CRTE arm compared to their values at V2, whereas in the placebo arm OxLDL levels were higher than their levels at V2 (Fig. 1). Significant differences between study arms were observed at 0, 180, 360, and 480 min. Furthermore, changes in AUC_0–480_ of OxLDL from V2 to V3 were significantly different between CRTE and placebo arms (*p*<0.0001).

**Table 2:** Postprandial OxLDL, glucose, and TG serum levels during the study

		Placebo			CRTE			
				
	Time	*n*	Mean±SEM	p* within treatment	*n*	Mean±SEM	p* within treatment	p* between treatments
OxLDL (U/L)								
V2	T0	71	62.30±25.57		75	63.30±27.75		0.821
	T180	71	53.66±18.69	<0.001	75	54.88±24.28	<0.001	0.7347
	T240	71	56.10±22.11	<0.001	75	57.21±27.44	<0.001	0.7898
	T360	71	55.31±23.05	<0.001	75	58.61±27.78	0.020	0.4376
	T480	71	59.20±22.62	0.060	75	59.12±27.27	0.015	0.9832
V3	T0	71	67.53±25.63		75	61.73±23.39		0.1547
	T180	71	64.95±26.95	0.218	75	58.85±27.32	0.030	0.1764
	T240	71	63.13±25.77	0.706	75	59.76±30.29	0.098	0.4713
	T360	71	65.79±27.01	0.084	75	58.85±25.06	0.015	0.1095
	T480	71	61.32±29.47	0.634	75	54.69±21.73	<0.001	0.1260
Glucose (g/L)								
V2	T0	71	0.91±0.07		73	0.90±0.07		0.6323
	T30	71	1.21±0.20	<0.001	72	1.16±0.19	<0.001	0.1210
	T60	71	1.02±0.27	<0.001	73	0.93±0.25	0.236	0.0455
	T90	71	0.85±0.21	0.018	73	0.83±0.24	0.005	0.5266
	T120	71	0.85±0.17	0.007	73	0.82±0.19	<0.001	0.3145
	T240	71	0.88±0.14	0.109	75	0.83±0.12	<0.001	0.0212
	T360	71	0.87±0.06	<0.001	75	0.88±0.09	0.044	0.6435
	T480	70	0.87±0.05	<0.001	74	0.87±0.06	<0.001	0.9076
V3	T0	71	0.91±0.07		75	0.89±0.07		0.1791
	T30	71	1.22±0.17	<0.001	74	1.16±0.18	<.001	0.0293
	T60	71	1.00±0.24	0.001	75	0.91±0.25	0.720	0.0237
	T90	70	0.85±0.19	0.023	75	0.80±0.17	<0.001	0.0595
	T120	71	0.87±0.16	0.086	75	0.81±0.16	<0.001	0.0172
	T240	71	0.86±0.10	0.001	74	0.85±0.12	<0.001	0.3805
	T360	71	0.88±0.06	<0.001	73	0.89±0.07	0.205	0.4851
	T480	71	0.89±0.08	0.068	75	0.88±0.06	0.101	0.8741
Triglycerides (g/L)								
V2	T0	71	0.86±0.37		73	1.01±1.49		0.4161
	T30	71	0.80±0.34	0.004	72	0.97±1.11	0.415	0.2142
	T60	71	1.01±0.40	<0.001	73	1.16±1.21	0.003	0.3133
	T90	71	1.17±0.63	<0.001	73	1.26±1.11	<0.001	0.5595
	T120	71	1.17±0.44	<0.001	73	1.32±1.05	<0.001	0.2625
	T240	71	1.16±0.51	<0.001	75	1.28±0.74	0.030	0.2274
	T360	71	1.02±0.48	<0.001	75	1.05±0.54	0.830	0.7469
	T480	71	0.78±0.35	0.016	74	0.77±0.41	0.142	0.8479
V3	T0	70	0.95±0.40		75	0.87±0.40		0.2381
	T30	71	0.95±0.43	0.057	74	0.83±0.39	0.263	0.0778
	T60	71	1.16±0.48	<0.001	75	1.02±0.43	0.920	0.0800
	T90	71	1.23±0.53	<0.001	75	1.14±0.49	0.388	0.2953
	T120	70	1.31±0.58	<0.001	75	1.24±0.49	0.146	0.4743
	T240	71	1.29±0.63	<0.001	74	1.26±0.62	0.121	0.7485
	T360	71	1.11±0.45	<0.001	74	1.09±0.52	0.688	0.8782
	T480	71	0.82±0.36	0.287	75	0.76±0.36	0.124	0.3250

STD, standard deviation; V, visit.

* *p* value by t-test indicates the statistical significance of the change within or between group.

### TG, glucose, and insulin levels after high-fat meal challenge

**Fig. 2:**
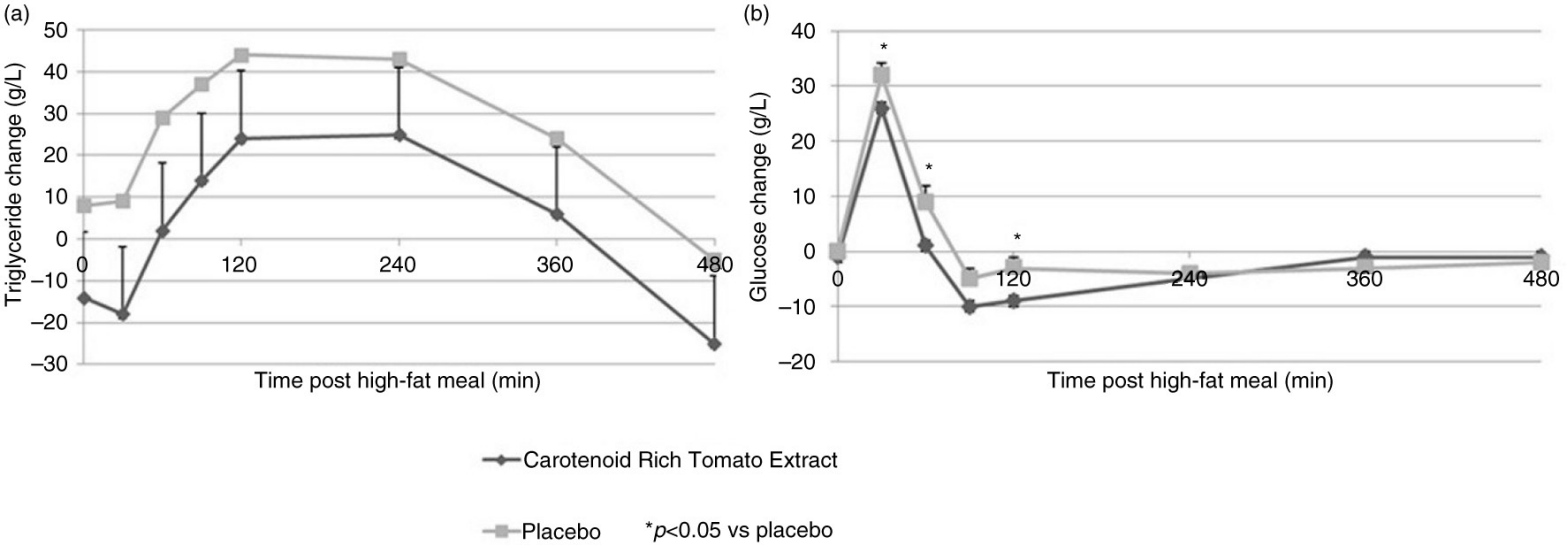
Change of postprandial plasma triglycerides (a) and glucose (b) levels at V2 to V3 by time points and treatment (mean±SEM).

Changes in TG and glucose serum levels after high-fat meal between V2 and V3 at different time points are shown in Fig. 2, and absolute values are reported in Table 2. Changes in TG response after the high-fat meal between V2 and V3 were not different between groups at any time point, and the change in TG AUC_0–480_ from V2 to V3 was close to significance (*p*=0.08). At V3, lowering of TG response in the CRTE arm compared to the placebo was close to significance at 30 and 60 min after the high-fat meal (*p*=0.08; Table 2). Changes in glycemic response were significantly improved at 30 and 60 min after a high-fat meal in the CRTE group compared to the placebo (Fig. 2b), whereas changes in glucose AUC_0–480_ from V2 to V3 were not different between groups (*p*=0.94). Glycemic response at V3 (absolute values; Table 2) was significantly lower at 30, 60, 90, and 120 min after the high-fat meal in the CRTE arm compared to the placebo (Table 2).

**Fig. 3:**
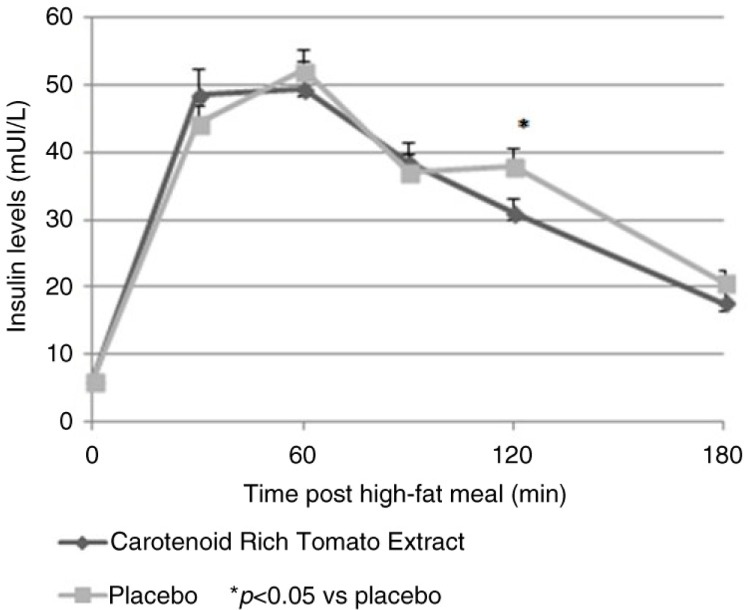
Change of plasma insulin levels at V3 by time points and treatment (mean±SEM).

Insulin levels measured at V3 (Fig. 3) were not lower in the CRTE group compared to the placebo, except at 120 min after the high-fat meal, as depicted in Fig. 3. Insulin AUC_0–480_ was not different between groups.

## Discussion

The effect of treatment with CRTE once daily for 2 weeks on postprandial OxLDL in healthy adult men and women with normal weight was evaluated in this single-center, randomized, double-blind, parallel-groups, placebo-controlled study. The power calculation, comparability of population features at baseline, low drop-out rate in the study, adequate compliance to intervention, and intention-to-treat statistical analysis support the validity of our trial results. The absence of follow-up on compliance to dietary and lifestyle recommendations is nevertheless a weakness of the study design.

Increased serum carotenoids at the end of intervention in the CRTE group witnessed the bioavailability of the product throughout the study period. This is in line with existing literature on tomato carotenoid bioavailability (20).

Concomitantly to this increase, the effects of 2-week supplementation with CRTE on OxLDL levels after a high-fat meal were evaluated. Reducing plasma OxLDL levels is considered a beneficial effect for atherosclerosis prevention and in the context of health claims for food products in the European Union (21). Interestingly, high inter-individual variability was observed in the OxLDL response to the high-fat meal, because only about one third of study population showed increased postprandial OxLDL levels. This explains why, on average, no significant increase in OxLDL levels was observed. The lack of deleterious effect of a high-fat meal on postprandial OxLDL levels was also reported in other studies. Reverri et al. (22) found no significant change in OxLDL concentration after consumption of a high-fat meal containing black beans or two control supplements. Another study (23) assessed the effect of a standardized high-fat meal on markers of inflammation and oxidative stress and found no significant changes in OxLDL concentrations in lean, obese non-diabetic, and type 2 diabetic men. A significant strength of this particular study included the evaluation of three well-defined patient populations and the use of a water control meal. In the current study, although a high-fat meal challenge did not exert the expected effect on metabolic response and flawed between-group comparisons in absolute OxLDL values at each visit, importantly the CRTE supplementation decreased changes in postprandial OxLDL levels for up to 8 h after ingestion of the high-fat meal, with a significant within treatment reduction in the CRTE, but not in the placebo group at 180, 360, and 480 min. Furthermore, when analyzing the change in OxLDL levels between V2 and V3 in the current study, the 2-week supplementation period resulted in a reduction of baseline levels (immediately before the meal) in the CRTE arm compared to placebo arm (*p*≤0.05). Although the elevation in baseline OxLDL values observed in the placebo group and not in the active treatment group at the V3 baseline is not fully understood, it can be hypothesized that this elevation was blocked in the active treatment group due to the anti-oxidative effect of the tested product (at this time point this group was already treated for 13 days). These results complete former observations from a pilot intervention trial (four healthy subjects) where a lycopene oleoresin administered before a high-fat meal challenge decreased LDL susceptibility to oxidation (24). Other plant-related substances and extracts have been reported to improve lipid peroxidation after either acute or chronic consumption, such as olive oil hydroxytyrosol (25, 26) or red wine polyphenols (27), but few have been investigated for their protective role after a high-fat meal challenge in healthy subjects. Additional studies should focus on acute and chronic effects of CRTE in normal life settings without metabolic challenge.

Changes observed in glucose, TG, and insulin values in the CRTE treatment arm (both in the change from baseline and AUC analysis) were not clinically meaningful by themselves, although together they may constitute an additive favorable effect of CRTE on metabolic outcomes after a high-fat meal. Longer exposure to treatment with CRTE may be required to see a more pronounced effect on the parameters assessed in the present study. The absence of insulin level measurements during the baseline visit did not allow us to draw a conclusion on the effect of the CRTE intervention on insulin response.

Additional information on the mechanisms of action of CRTE and its metabolic effects in response to a high-fat meal challenge could have been collected through analysis of inflammatory markers such as interleukin 8 (IL-8), a chemokine that has been recently described as an early marker of endothelial stress (15).

Results support safety and tolerance of CRTE in healthy normal weight subjects. No significant laboratory abnormalities or clinically significant changes in vital signs or physical finding were reported during the study.

## Conclusions

Overall, the results of this study indicate that CRTE taken once daily for 2 weeks has a favorable effect on postprandial LDL oxidation, glucose, insulin, and TG levels for up to 8 h. CRTE was well tolerated throughout the study period, and additional trials are needed to prove the repeatability of these results in other sub-populations such as subjects at-risk of cardiovascular diseases.
